# Application of a Quantitative Real-Time PCR Assay for Early Detection of *Salmonella enterica* Serovar Enteritidis on Poultry Farms During an Outbreak in New South Wales, Australia (2018–2020)

**DOI:** 10.1155/tbed/9937941

**Published:** 2025-06-04

**Authors:** Emily Onizawa, Mark E. Westman, Daniel R. Bogema, Ania T. Deutscher, Kieran Eamens, Melinda L. Micallef, Tammy McDonogh, Cheryl Jenkins

**Affiliations:** ^1^NSW Department of Primary Industries and Regional Development, Elizabeth Macarthur Agricultural Institute, Menangle, NSW 2568, Australia; ^2^Sydney School of Veterinary Science, The University of Sydney, Sydney, NSW 2006, Australia; ^3^Sydney Institute for Infectious Diseases, The University of Sydney, Sydney, NSW 2006, Australia

**Keywords:** biosecurity, diagnosis, food safety, molecular, poultry, public health, salmonella enteritidis, veterinary science, zoonotic

## Abstract

*Salmonella* spp. are a significant cause of human foodborne illness globally, with ingestion of contaminated eggs a major vehicle for infection. *Salmonella enterica* serovar Enteritidis (*S*. Enteritidis, SE) is the serovar most linked to egg-related foodborne salmonellosis in most developed countries. Until 2018, the Australian egg industry was considered free of SE. This report documents the diagnostic testing performed on samples from egg layer farms across New South Wales (NSW), Australia, as part of a SE outbreak response between 2018 and 2020. Testing was undertaken following a cluster of cases of SE infection in humans traced to the consumption of eggs originating from a single contaminated poultry farm. Quantitative real-time polymerase chain reaction (qPCR) testing was used to screen environmental and animal samples (*n* = 2058) from 29 different properties identified through contact tracing. Confirmatory bacterial culture (*n* = 717) was performed on any SE qPCR-positive samples and a subset of qPCR-negative and qPCR-inconclusive samples. In total, 13/29 (45%) of egg layer farms were SE-positive by qPCR testing, with 12/13 (92%) of these farms confirmed SE-positive by bacterial culture and serotyping. Both environmental and animal samples produced SE-positive results, in particular surface swabs, boot covers, feces, and eggs. When qPCR testing and bacterial culture were performed side-by-side, qPCR testing to detect SE compared to bacterial culture had sensitivity of 100% (43/43) and specificity of 94.1% (238/253; 95% confidence interval[CI] 91.4–96.8). SE isolates obtained during the outbreak were predominantly phage type (PT)1b and PT12. Whole genome sequencing (WGS) of SE isolates from 9 of 12 culture-positive properties confirmed that they were all sequence type 11, Clade B, and derived from a single source. As a result of rapid qPCR detection of SE on contaminated farms, appropriate biosecurity responses were implemented, and NSW commercial layer farms were again considered SE-free in August 2020. This report highlights the utility of high-throughput molecular testing for SE in outbreak situations.

## 1. Introduction

Salmonellosis, caused by infection with non-typhoidal *Salmonella*, is one of four key bacterial causes of diarrhoeal diseases in people globally, along with infection from Shiga toxin-producing *E. coli*, cholera (caused by *Vibrio cholerae*), and campylobacteriosis (usually caused by *C. jejuni* or *C. coli*) [1]. Although most cases of salmonellosis in people are mild and self-limiting, dehydration caused by severe vomiting and diarrhea can sometimes be life-threatening. Non-typhoidal salmonellosis is responsible for more than 150 million cases of gastroenteritis and 57,000–155,000 deaths annually [2, 3]. A recent global systematic review and meta-analysis estimated the overall mortality for non-typhoidal salmonellosis to be 15% [4]. In Australia, non-typhoidal *Salmonella* spp. infections are a leading cause of gastroenteritis-associated hospitalization and death in people [5]. In 2023, the estimated cost of non-typhoidal salmonellosis and its sequelae in Australia was AUD $161 million (representing 5.7% of the total cost of foodborne disease to Australia), comprised mainly of health care costs, costs associated with lost productivity, and premature mortality [6, 7].

Of the non-typhoidal *Salmonella* species, *Salmonella enterica* serovar Typhimurium (*S*. Typhimurium, ST) and *S. enterica* serovar Enteritidis (*S*. Enteritidis, SE) are the two serovars (serotypes) found in industrialized countries to most commonly cause severe gastroenteritis in humans, usually following consumption of contaminated food products [8–14]. In one report of 37 countries that participated in a World Health Organisation (WHO) *Salmonella* monitoring scheme, the overall proportion of *Salmonella* infections caused by ST and SE during a 7-year period (2001–2007) were 17% and 45%, respectively [8]. The overall number of cases of gastroenteritis caused by SE has increased markedly since the 1970s and 1980s and is linked to a sharp rise in the prevalence of this serovar in poultry [8, 9, 14, 15]. While improved cleaning practices have decreased the number of human cases acquired through eggshell contamination, cases resulting from internally contaminated eggs are on the rise in many countries [16]. In contrast, the proportion of human infections caused by SE in some European countries has decreased since the late 1990s due to the introduction of a SE vaccine in poultry [17–19].

In contradistinction to most other developed countries, ST (not SE) is the predominant serovar in Australia identified in cases of human non-typhoidal salmonellosis [20]. ST is responsible for one quarter to one half of all reported human salmonellosis infections annually in Australia [8, 21, 22]. Other *S. enterica* serovars commonly isolated from human clinical cases in Australia include Anatum, Birkenhead, Bovismorbificans, Chester, Heidelberg, Infantis, Muenchen, Saintpaul, Wangata, and Virchow [8, 21, 23]. Clade A and Clade C strains of SE are endemic in the state of Queensland, likely have environmental niches, and do occasionally cause sporadic cases of salmonellosis in people [8, 21, 24–26]. However, human cases of SE infection in Australia, usually caused by Clade B strains, are almost always acquired overseas [23]. As of June 2017, the Australian egg industry was considered to be free of SE [27].

The aim of the current study is to report and describe the molecular and bacterial testing of poultry farms undertaken in response to the first Clade B outbreak of SE in domestic poultry farms in New South Wales (NSW; Australia's largest state) in 2018–2020 and recommend a rapid diagnostic approach for laboratories involved with ongoing surveillance of the poultry industry in the future.

## 2. Materials and Methods

### 2.1. In-House Verification of the SE qPCR Assay Used

A multiplex quantitative real-time polymerase chain reaction (qPCR) assay previously validated for the environmental detection of *Salmonella* in food production facilities was applied in this study. The SE qPCR assay used was previously validated with a panel of 329 isolates including 126 serovars of *Salmonella* [28]. Additionally, we conducted in-house verification of the sensitivity and specificity of the assay using DNA extracted from a total of 76 isolates including 46 SE isolates (of which four were human isolates from this outbreak, and two Clade A isolates; kindly supplied by Institute of Clinical Pathology and Medical Research [ICPMR], Westmead Hospital), 23 isolates of different (non-SE) *Salmonella* serovars, and seven isolates representing other Enterobacterales (Supporting Information 1: Table S1).

### 2.2. Initial Identification of SE Cases in People in NSW

In July and August of 2018, routine whole genome sequencing (WGS) of cases of human salmonellosis in NSW by health authorities identified a cluster of SE infections (phage type[PT] 7a) linked to the consumption of a frozen meringue cake (Craig Shadbolt, Biosecurity & Food Safety, NSW Department of Primary Industries and Regional Development, personal communication). In turn, public health officials were able to trace the source of the eggs used for the cake to a commercial egg production facility in south-western Sydney, NSW, and environmental samples from this farm tested SE-positive [29–31]. From September 2018 to March 2020, samples from egg layer farms in Sydney were sent to Elizabeth Macarthur Agricultural Institute (EMAI), Department of Primary Industries and Regional Development (DPIRD), for testing to determine the extent of the spread of SE in NSW layer farms. Farms tested consisted of infected premises, trace premises and dangerous contact premises. Testing of grading facilities and farms that had been decontaminated was also undertaken.

### 2.3. Sample Population

Various sample types were collected for testing according to a defined sampling schedule [32], including both environmental and animal (poultry) samples (Table 1). Environmental samples routinely submitted included boot covers (worn while walking through a shed), and surface swabs from walls, floors, egg processing equipment such as conveyer belts and processing tables, cleaning equipment including dustpans and brooms, vehicle tires, door handles, gates, fans, perches, hutches, feeding boxes and drinking stations. Environmental samples were collected from specific areas and labeled accordingly (e.g., Shed 1 Row 1), and results reported according to the individual area or shed that the sample was collected from to allow accurate identification of contaminated sites on the property. Animal (poultry) samples routinely submitted included eggs and feces. Poultry fecal samples were collected from the floor of the shed and represented multiple individuals. Some nonroutine samples were also opportunistically collected and tested including dirt/soil or dust, water, organ and heart blood swabs from dead poultry, rodent carcasses, and rodent and cockroach feces.

### 2.4. Initial Sample Handling and Preparation for Testing

Environmental samples did not require preprocessing. The egg samples were pooled (six eggs per pool) and homogenized by cracking the eggs into a sterile container and thoroughly shaking (shells included).

**Figure 1:**
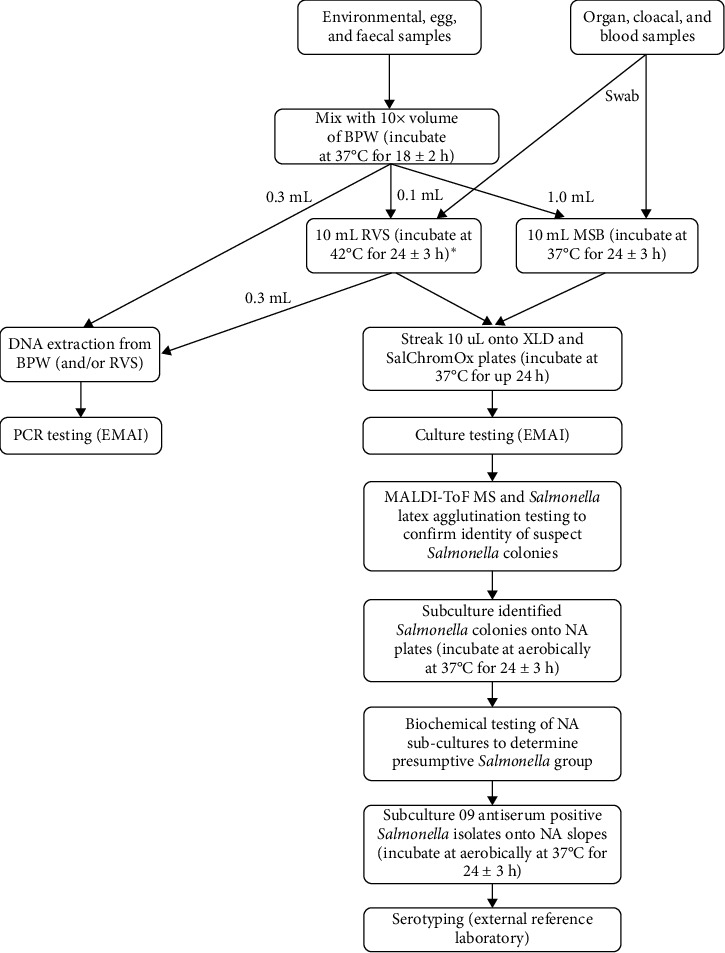
Approach to testing samples at the Elizabeth Macarthur Agricultural Institute (EMAI) collected from poultry layer farms in New South Wales (NSW), Australia. Refer to Table 1 for a full description of sample types. BPW, buffered peptone water; MALDI-ToF MS, matrix-assisted laser desorption/ionization-time of flight mass spectrometry; MSB, mannitol selenite broth; NA, nutrient agar; RVS, Rappaport Vassailiadis soya peptone broth; SalChromOx, *Salmonella* chromogenic agar; XLD, Xylose Lysine Decarboxylase agar.  Figure 1 made with diagrams.net Flowchart Maker and Online Diagram Software. *⁣*^*∗*^Minimum 16 h for molecular testing.

Following any initial preprocessing, all samples aside from tissue swabs were placed into a screw-top tube containing 10 × volume of buffered peptone water (BPW; Thermo Fisher Scientific, Waltham, MA, USA, Cat. No. CM0509R) and incubated at 37°C for 18 ± 2 h. For selective *Salmonella* enrichment, 0.1 mL of incubated BPW was transferred into 10 mL of prewarmed Rappaport Vassiliadis soya peptone broth (RVS; Edwards Group, Narellan, NSW, Australia, Catalog No. 4081) and incubated at 42°C for 24 ± 3 h. Additionally, 1.0 mL of incubated BPW was then transferred into 10 mL of prewarmed mannitol selenite broth (MSB; Edwards Group, Cat. No. 4084) and incubated at 37°C for 24 ± 3 h. Swabs from animal tissues were placed directly into 10 mL of RVS and MSB and incubated under the same conditions. 1 mL aliquots of BPW, RVS, and MSB samples were frozen at −20°C after the required incubation for DNA isolation later, if required.  Figure 1 summarizes the approach taken to handling and processing samples from NSW layer farms for molecular testing and bacterial culture.

### 2.5. Molecular Testing

In all cases, SE qPCR was initially performed on incubated BPW samples. 90 µL of DNA was eluted from an extraction of 300 µL of BPW using the MagMAX Core Nucleic Purification Kit (Thermo Fisher Scientific, Cat. No. A32702), following the manufacturer's instructions, on the KingFisher Flex Purification System. When required (e.g., due to inconclusive results or PCR inhibition, discussed later), DNA was also extracted from RVS following the same protocols. RVS enrichment extractions demonstrated greater sensitivity than repeat extraction of BPW, with RVS also favored over MSB enrichment due to higher recovery of the *invA* target (data not shown). Prior to extraction, 2 µL of VetMax Xeno Internal Positive Control DNA (Thermo Fisher Scientific, Cat. No. A29762) was added to each sample (i.e., incubated BPW or RVS) to monitor for any inhibition during qPCR testing.

qPCR testing was performed with a final reaction volume of 20 µL comprising 2 µL extracted DNA, 10 µL TaqMan Environmental Master Mix 2.0 (Applied Biosystems, Thermo Fisher Scientific, Cat. No. 4396838), 0.8 µL VetMax Xeno Internal Positive Control-LIZ or VIC assay (Thermo Fisher Scientific, Cat. No. A29766 or A29767), and final concentrations of primers and probes as reported. The concentrations of primers and probes used in the qPCR assay were as follows: 200 nM *invA* forward and reverse primers and 30 nM probe; 250 nM *prt* forward and reverse primers and 200 nM probe; and 250 nM *Sdf-1* forward primer, 300 nM *Sdf-1* reverse primer, and 40 nM probe [28]. A negative control (molecular grade water, MGW), positive plasmid standard dilution series in tRNA and a 1/10 dilution of VetMax Xeno Internal Positive Control DNA were included in each qPCR run. Negative process controls of BPW and RVS (when relevant) were also set up with each submission and used as negative extraction controls. Cycling conditions were 95°C for 10 min for 1 cycle, followed by 45 cycles of 95°C for 15 s and 60°C for 60 s. All reactions were run on an Applied Biosystems 7500 Fast Thermocycler or a Quantstudio 5 Real-Time PCR System (Thermo Fisher Scientific).

The qPCR assay used simultaneously targets *Salmonella* spp. (*invA* gene), Group D serovars of *S*. *enterica* which includes SE (*prt* gene; also detects Group A *Salmonella* spp.), and the SE serovar (*Sdf-1* gene) [28]. The limits of detection (LOD) for each target (*invA*, *prt* and *Sdf-1*) with 95% confidence were 5.20, 5.18, and 11.18 genes copies, respectively. PCR threshold targets were set to 0.01, 0.1, and 0.02 *∆*Rn for *invA*, *prt* and *Sdf-1*, respectively, and Xeno set to auto-threshold prior to analysis. Regardless of amplification for other targets, samples with a Xeno cycle threshold (Ct) value >33 were recorded as inconclusive.

Samples with no amplification in any of the *Salmonella* targets were recorded as negative. Samples that amplified all three targets (i.e., *invA*, *prt*, and *Sdf-1*) were deemed positive for SE. Samples that amplified *invA* only were reported as positive for *Salmonella* spp. (i.e., indicated the presence of non-SE *Salmonella* serovars). Samples with any other amplification combination, or inconclusive due to Xeno Ct value >33, were recorded as inconclusive. Inconclusive samples were resolved either by repeat extraction of the incubated BPW or DNA extraction from the incubated RVS enrichment and repeat qPCR testing.

During the initial stages of the outbreak, all samples that were qPCR positive for Group D *Salmonella* (i.e., *invA* and *prt* positive) were subject to *Salmonella* culture for the confirmation of viable SE organisms. From May 2019 onwards, all *invA* positive samples were cultured to assist with the identification of other *Salmonella* serovars present on NSW farms.

### 2.6. Sanger Sequencing for Confirmation of qPCR Positive Samples and Isolates

Select qPCR-positive samples or bacterial isolates were amplified using conventional PCR and subjected to Sanger sequencing to confirm that the three target genes (in particular *Sdf-1*) had been amplified. qPCR primers were applied in a conventional PCR format (without the addition of probes) including volumes of 1 × BioTaq polymerase and 1 × BioTaq buffer (Bioline, Memphis, TN, USA, Cat. No. BIO-21040) in a final reaction volume of 25 µL. The QIAquick PCR Purification Kit (Qiagen, Hilden, Germany, Cat. No. 28104) was utilized to purify positive amplicons before being sent for Sanger sequencing at the Australian Genome Research Facility (Westmead, Sydney). Sequencing analysis was conducted using Geneious Prime vR11 (Biomatters, Auckland, NZ).

### 2.7. Bacterial Culture, Identification, Serotyping and Phage Typing (PT)

Bacterial culture was performed on a subset of samples as a confirmatory test for samples testing SE qPCR-positive, qPCR-positive for *Salmonella* spp., or qPCR-inconclusive with initial PCR testing, and to enable direct comparison of qPCR testing and bacterial culture for SE detection. Performing *Salmonella* culture on initially qPCR-inconclusive samples helped with turnaround time for culture results and workflow if the sample was *Salmonella* qPCR-positive with repeat testing.

For all types of samples, 10 µL of RVS broth and 10 µL of MSB broth were cultured onto both Xylose Lysine Decarboxylase agar (XLD) plates made in-house and *Salmonella* chromogenic agar (SalChromOx) plates either made in-house or purchased (Thermo Fisher Scientific, Cat. No. PP2269). All plates were incubated under strict aerobic conditions at 37°C for 24 h and examined for growth. Suspect *Salmonella* colonies were identified as *Salmonella* spp. by matrix-assisted laser desorption/ionization-time of flight (MALDI-ToF) mass spectrometry using a MALDI Biotyper (Bruker, Billerica, MA, USA). Colonies identified as *Salmonella* spp. were subcultured onto nutrient agar (NA) plates (Thermo Fisher Scientific) and incubated aerobically at 37°C overnight, followed by confirmatory *Salmonella* latex agglutination testing (Thermo Fisher Scientific, Cat. No. DR1108A) and presumptive *Salmonella* serotyping with 09 polyvalent antiserum (Remel Europe, Dartford, UK, Cat. No. R30858201). A random selection of suspect *Salmonella* colonies was presumptively serotyped per sample (usually up to five) across the four *Salmonella* selective plates, as per the International Standard (ISO 6579-1:2017), the Australian Standard (AS 5013.10—2009), and the World Organisation for Animal Health recommendations [33, 34]. Occasionally, when only non-SE *Salmonella* colonies (i.e., 09 antiserum negative) were isolated from SE qPCR-positive samples, additional colonies (up to 15–20 colonies per sample) were screened.

Confirmed 09 antiserum positive *Salmonella* isolates were subcultured onto NA slopes, incubated aerobically at 37°C overnight, and sent for definitive serotyping at an external laboratory (Australian Salmonella Reference Centre, Institute of Medical and Veterinary Science, Adelaide; or NSW Enteric Reference Laboratory, Westmead). The reference laboratories also determined and reported the Kauffmann–White antigenic structure of the SE isolates submitted. From May 2019, it was decided to also send at least one *Salmonella* isolate per sample for serotyping by the NSW Enteric Reference Laboratory to investigate what other (non-SE) *Salmonella* serovars were present in NSW poultry environments. PT of a subset of SE isolates was performed by the Australian Salmonella Reference Centre, Adelaide, according to the Colindale PT scheme (Helen Hocking, South Australia Pathology, personal communication).

### 2.8. Genome Sequencing of Salmonella Isolates

Further characterization of SE isolates was performed by WGS using an Ion Torrent S5 (Thermo Fisher Scientific). DNA was extracted from pure *Salmonella* isolates using the KingFisher Flex Purification System (Thermo Fisher Scientific) or DNeasy Blood and Tissue Kit (Qiagen, Hilden, Germany, Cat. No. 69504) and eluted in 90 µL MGW prior to quantification on a Qubit Fluorometer (Thermo Fisher Scientific). Ion Torrent libraries were prepared, amplified, and quantified as previously described [35]. The quantified libraries were diluted to a concentration of 100 pM and loaded onto an Ion 530 chip using the Ion Chef system. The prepared chip was then run on the Ion Torrent S5 next generation sequencing system.

Raw sequencing reads were quality checked with fastp v 0.23.2 [36]. Preliminary analysis of completeness, phylogeny, and gene content was first performed using nullarbor v 2.0.20191013 [37], with modification of read files to a simulated paired-end format performed by a customized script (github) [38]. Assemblies generated by skesa v 2.4.0 [39] within nullarbor were then used to identify a suitable reference sequence with ReferenceSeeker v 1.8.0 [40]. Output from ReferenceSeeker was used to identify a reference sequence that maximized genome identity and conserved DNA in subsequent alignment generation and phylogenetic analysis. Sequencing reads and representative public SE complete assemblies were then aligned to the most suitable reference, *Salmonella* Enteritidis str. 2017K-0021 using Snippy v 4.4.3 (within the nullarbor package) [37]. A core genome alignment was then generated using snippy-core and SNP distances calculated using snp-dists v 0.8.2 (within the nullarbor package) [37]. Phylogenetic trees were inferred using IQ-TREE v 2.2.0.3 [41] with automated model selection [42]. Branch support values were calculated using the ultrafast bootstrap method within IQ-TREE [43]. Phylogenetic trees were rooted using the minimum variance method [44].

### 2.9. Statistical Analysis

Test outcomes were compared between sample types using Fisher's exact test. Test sensitivity for SE qPCR was calculated using the formula (sensitivity = number of true-positives/[true-positives + false-negatives] x100), and test specificity was calculated using the formula (specificity = number of true-negatives/[false-positives + true-negatives] ×100), using bacterial culture as the true result. The 95% confidence intervals (CI) were calculated using Microsoft Excel (Microsoft, Redmond, WA, USA). Statistical significance was considered at *p* < 0.05.

## 3. Results

### 3.1. In-House Verification of the SE qPCR Assay Used

None of the non-*Salmonella* Enterobacterales tested had any amplification with qPCR testing. Of the 23 non-SE *Salmonella* tested, all isolates amplified in the *invA* target, with no amplification in the *prt* and *Sdf-1* targets, except for *Salmonella* Dublin, which also yielded expected amplification in the serovar D-specific *prt* target. Of the 46 SE isolates tested, 44 tested SE qPCR-positive including four SE strains isolated from humans during the outbreak, yielding a diagnostic sensitivity of 95.7% (44/46). Interestingly, the two SE strains that failed to amplify in the *Sdf*-1 target (i.e., amplified in the *invA* and *prt* targets but not *Sdf-1*) represented Clade A strains. However, given the specificity of the assay (30/30, 100%) and the ability of the assay to amplify outbreak-associated (non-Clade A) SE strains, this assay was deemed suitable for use in this outbreak investigation.

### 3.2. Overall Testing Summary

**Figure 2:**
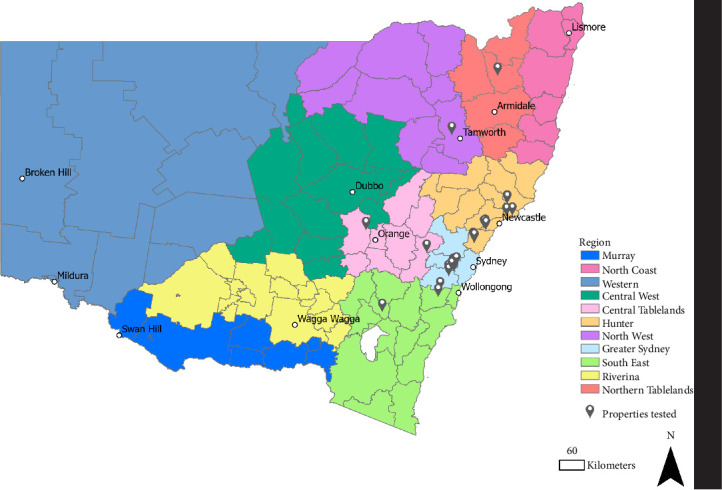
Samples from 29 egg layer farms located across New South Wales (NSW; see flags) were sent to Elizabeth Macarthur Agricultural Institute (EMAI) for SE testing. Any farms with direct links to a confirmed SE-positive farm had environmental samples collected for SE testing. The 11 NSW Government regions are shaded in different colors (see Legend). Map created using ArcGIS Pro 3.0.2.

A total of 46 submissions from 29 different egg layer farms or grading facilities in NSW were received at EMAI for testing during the outbreak (Figure 2).

Thirteen of 29 NSW egg layer farms (45%) were found to be SE-positive by qPCR testing. Of these 13 qPCR-positive properties, 12 (92%) had SE confirmed by bacterial culture and serotyping. The one property that returned SE qPCR-positive/culture-negative results was sampled on three different occasions; on the second and third submissions, the property tested SE qPCR-negative/culture-negative. Of the 16 SE qPCR-negative properties, 10 properties tested culture-negative, and six properties did not have bacterial culture performed.

Of the 23 properties that had bacterial culture performed, nine (39%) were positive for both SE and non-SE *Salmonella*, three (13%) were positive for SE only, 10 (43%) were positive for non-SE *Salmonella* only, and only one property (4%) was completely negative for all *Salmonella* spp. (culture was performed on all samples for this property for qPCR verification).

In total, 2058 individual samples were submitted to EMAI for SE testing, comprising 1609 environmental samples and 449 animal (poultry) samples (Supporting Information 2: Table S2). Since samples were almost always tested by qPCR first, and bacterial culture was predominantly performed to confirm qPCR-positive results, there was a higher amount of molecular testing (*n* = 2058) compared to culture testing (*n* = 717).

### 3.3. Molecular Results

SE was detected in 194/2058 (9%) samples by qPCR testing, while 280/2058 (14%) samples were qPCR-positive for other (non-SE) *Salmonella* serovars. Of the SE-positive samples, 157/194 (81%) were environmental samples and 37/194 (19%) were animal (poultry) samples. Environmental samples also represented most of the non-SE positive samples (254/280; 91%).

There was no difference between environmental and animal (poultry) samples with regards to the likelihood of a SE-positive result by qPCR testing (157/1609 versus 37/449; *p*=0.36; Fisher's exact test). Surface swabs were the most useful environmental sample tested to detect SE, with 126/1300 (10%) of samples qPCR SE-positive, comprising 126/157 (80%) of all qPCR SE-positive environmental samples. Feces was the most useful animal sample type tested to detect SE, with 20/145 (14%) of fecal samples testing qPCR SE-positive, comprising 20/37 (54%) of all qPCR SE-positive animal samples (Supporting Information 2: Table S2).

Repeat qPCR testing was performed on 173/2058 (8%) of samples due to either inhibition of samples (as identified by late internal control amplification; Xeno Ct value >33; *n* = 113) or inconclusive results (amplification of *prt* and/or *Sdf-1* without amplification of the *invA* target; *n* = 60). Of the samples requiring repeat qPCR testing due to inhibition, egg samples were most frequently represented (59/113; 52%), constituting 33% of all egg samples tested (59/181). Surface swabs were next most represented (33/113; 29%), constituting 2.5% of all surface swabs tested (33/1300), followed by fecal samples (20/113; 18%), constituting 14% of all feces tested (20/145). Samples requiring repeat qPCR testing were resolved by either repeat extraction of the incubated BPW (63/173; 36%) or extraction from the secondary RVS enrichment and repeat qPCR testing (110/173; 64%).

Sanger sequencing of qPCR amplicons on a subset of samples confirmed all three *Salmonella* targets (i.e., *invA*, *prt*, and *Sdf-1* genes) amplified as expected.

### 3.4. Bacterial Culture Results

A total of 717 samples were tested by bacterial culture. Of these, 421 (59%) samples were tested to confirm a qPCR-positive result or to investigate a qPCR-inconclusive result, and 296 (41%) samples were cultured regardless of the qPCR result to enable direct comparison of bacterial culture and qPCR results and/or to determine *Salmonella* presence following postmortem examination.

In total, 104/717 (15%) samples were confirmed to be SE-positive by culture, and 270/717 (38%) samples were culture positive for other (non-SE) *Salmonella* types. Of the SE culture-positive samples, 82/104 (79%) were environmental samples and 22/104 (21%) were animal (poultry) samples. Environmental samples represented most of the non-SE culture-positive samples (242/270; 90%).

There was no difference between environmental and animal (poultry) samples with regards to the likelihood of a SE-positive result by bacterial culture (82/562 versus 22/155; *p*=0.54; Fisher's exact test). Surface swabs produced the highest number of SE isolates from environmental samples (55/82; 67%); however, culture of boot covers returned a significantly higher rate of SE-positive results than culture of surface swabs (25/109 [23%] vs., 55/422 [13%]; *p*=0.016; Fisher's exact test). As for molecular testing, feces was the most useful animal sample type tested to detect SE, with 9/62 (15%) of fecal samples testing culture SE-positive. Of the 22 animal samples that were SE culture-positive, nine samples (41%) were feces (Supporting Information 2: Table S2).

From a total of 409 *Salmonella* isolates cultured, 381 were sent for serotyping. Thirty-four different *Salmonella* serovars (serotypes) were identified, including those typed as non-SE subsp. 1, but not typed further (Table 2). Other commonly isolated *Salmonella* serovars included Infantis (74/381, 19.4%) from six different properties and Typhimurium (38/381, 10.0%) from two different properties. When SE was present, nine of the 12 properties (75%) presented with other serovars of *Salmonella* as well. When multiple *Salmonella* colonies (i.e., two or more) isolated from the same sample were serotyped, different serovars were obtained on 13/29 (45%) occasions, all from non-poultry (environmental) samples. ST was the most identified serovar in these multiple serovar samples (9/13, 69%; Supporting Information 3: Table S3). Different *Salmonella* serovars were never identified from a single animal sample.

RVS broth outperformed MSB for obtaining both SE and non-SE *Salmonella* isolates. In a subset of samples when results from RVS and MSB were compared side-by-side, 39/70 culture-positive samples across three different submissions only had *Salmonella* growth in the RVS broth and not MSB.

The Kauffmann–White antigenic structure of all 104 SE isolates recovered was 9,12:g, m:-, consistent with the SE formula listed by the WHO Collaborating Centre for Reference and Research on *Salmonella* (WHO Collaborating Centre, Pasteur Institute, Paris, France) [45].

Of the 104 SE isolates recovered, a subset (*n* = 16) was sent for PT. Isolates from samples received in 2019 were predominantly PT12, while most isolates tested in 2020 were PT1b, with one property having both PT1b and 12 isolated from the same submission in 2020 (Property 24). However, only isolates from six different SE-positive properties were sent for PT, and half of isolates with PT performed were from the one property (Property 6), therefore definitive conclusions from this data are difficult to make (Table 3).

### 3.5. Comparison of qPCR and Bacterial Culture Results for SE Testing

Table 4 shows the results of the 296 samples tested concurrently with qPCR and bacterial culture to enable a direct comparison. Results from samples not deliberately tested side-by-side (i.e., culture performed to confirm a qPCR-positive result or to investigate a qPCR-inconclusive result) were excluded from analyses to remove testing bias. The samples tested were collected from nine different properties, including five SE culture-positive properties. Compared to the culture result, qPCR testing to detect SE had sensitivity of 100% (43/43) and specificity of 94.1% (238/253; 95%CI 91.4–96.8). Supporting Information 4: Table S4 lists the details of the 15 discordant results (SE qPCR-positive, SE culture-negative; 13 environmental samples and 2 animal samples [poultry feces]).

### 3.6. Genotyping of Salmonella Enteritidis Isolates

**Figure 3:**
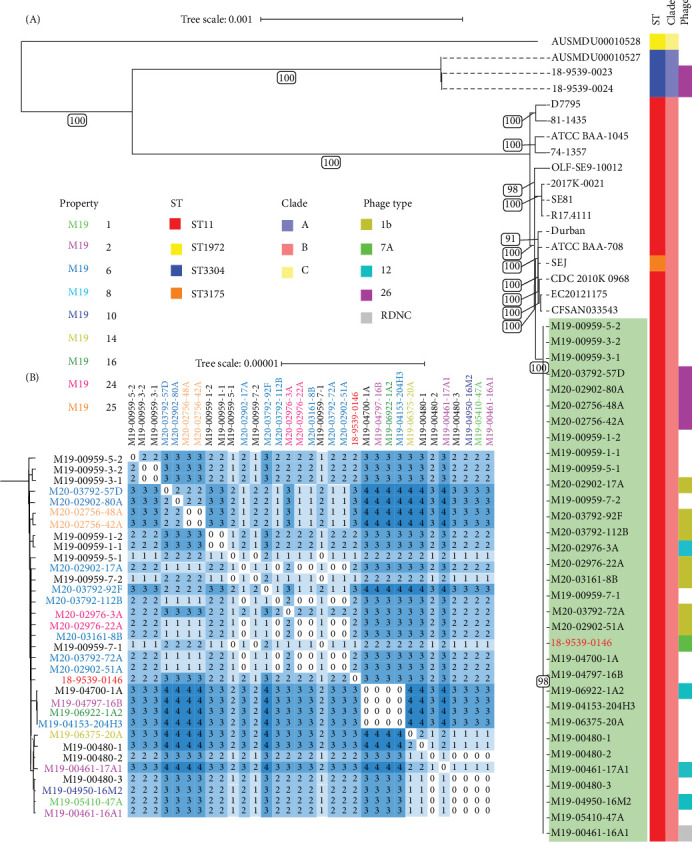
(A) core genome SNP phylogenetic tree generated from 33 Clade B *S. enterica* serovar Enteritidis (SE) isolates sequenced during the New South Wales 2018–2020 outbreak (highlighted in green, including the index isolate sourced from frozen meringue cake in red text), and representative complete SE genomes from Clades A, B and C. Sequence type (ST), clade ID, and phage type are shown with colored strips to the right of the tree. Branch support values were calculated using the UFBoot method [43] with 1000 replicates, with values above 90 shown in the figure. (B) pairwise heatmap showing number of core-genome SNP differences between 33 Clade B *S. enterica* serovar Enteritidis (SE) isolates sequenced during the New South Wales 2018–2020 outbreak. Isolates obtained at EMAI and sequenced in this study (*n* = 20) are in colored text according to the nine different properties they were derived from, while isolates received from other laboratories during the outbreak and sequenced (*n* = 12) are colored black. The index isolate sourced from frozen meringue cake is colored in red text (18-9539-0146). EMAI, Elizabeth Macarthur Agricultural Institute.

WGS analysis was performed on 20/104 (19%) of SE isolates sourced during the 2018–2020 outbreak, obtained from 9 (of 12) different SE culture-positive properties. To determine if they were linked by a common source, a subset of SE isolates derived from environmental and animal samples were tested. In addition, 12 SE isolates obtained from other laboratories during the outbreak also had WGS performed, as well as the index SE isolate sourced from frozen meringue cake (Supporting Information 5: Table S5). All raw reads used in this study passed quality checks with all isolates showing ≥88% of reads at Q20 and ≥50% of reads at Q30. Average coverage depth ranged from 33.5x to 109x and 94.4% to 96.9% of the reference sequence (including chromosomes and plasmids) was covered at 10x depth. Phylogenetic analysis of core-genome alignments placed all outbreak isolate sequences as sequence type (ST) 11 within a single monophyletic clade with strong (>90) bootstrap support (Figure 3A). All SE isolates from the outbreak were Clade B, genetically distinct from Clade A and Clade C isolates previously isolated from Queensland, Australia (Figure 3A). Further analysis of SNP distances showed a maximum pairwise distance of 4 SNPs between SE isolates collected in 2019 and 2020 (Figure 3B), confirming a single outbreak cluster according to previously established criteria (isolate genome distance <21 SNPs) [46].

## 4. Discussion

SE was identified at 13 separate properties in the greater Sydney region by molecular testing over an 18-month period during an outbreak in NSW, Australia in 2018–2020. Almost all (12/13) of these properties had qPCR results confirmed by bacterial culture and *Salmonella* serotyping. The 12 infected premises had an individual decontamination plan created by an expert veterinary consultant, approved by the NSW Chief Veterinary Officer (CVO), and carried out by authorized officers. For an infected premise to be cleared of SE infection, repeat sampling based upon earlier sampling activities was carried out. Ultimately, the CVO approved any application for a property status to be changed from SE-infected to SE-resolved. The one property with discordant results (qPCR-positive/culture-negative) most likely had very low levels of SE and/or non-viable SE. Fortunately, despite not being destocked, and thorough decontamination only occurring as flocks came to end of life, this property on later sampling visits tested SE qPCR-negative. We believe this property was a trace property with minor SE contact from packaging or a transport vehicle, leading to non-viable SE being detected by molecular testing (Craig Shadbolt, personal communication). As a result of the approach outlined in this manuscript for rapid molecular diagnosis of SE, and subsequent management decisions that facilitated containment and decontamination of SE-positive properties, NSW was again considered SE-free in August 2020 [47]. This is the first time a case cluster of human SE infections in Australia has been linked to the presence of SE on poultry farms and demonstrates that SE represents a major emerging public health risk to poultry workers and consumers in NSW.

Following the detection of SE on NSW poultry farms in 2018, a *Salmonella* Enteritidis Biosecurity Control Order was issued in 2019 (amended in 2020, now in effect until 30 June 2025) in pursuance of section 62 of the *Biosecurity Act 2015* [48]. The Control Order mandated that the person/s in charge of a licensed egg business must follow guidelines to minimize the risk of SE spread including improved biosecurity measures (e.g., clean boots, handwashing, vehicle control and disinfection of waste), and pursue mandatory SE testing by an accredited laboratory. Australian commercial egg producers were advised that the easiest way to comply with the Control Order was to participate in the voluntary National *Salmonella* Enteritidis Monitoring Accreditation Program (NSEMAP) [49], or collect environmental samples from every shed every 12–15 weeks and retain test results for auditing purposes for at least 24 months [32, 50]. The Biosecurity Control Order did not mandate the type of diagnostic testing to be performed by accredited laboratories. Based on the results of the current study, EMAI's ongoing approach to SE surveillance is to perform molecular testing of all environmental samples and to only pursue bacterial culture for confirmation of qPCR-positive results.

In future, (i.e., beyond the SE Control Order), it is likely surveillance and regular testing of samples for SE from all egg-layer farms will need to continue to facilitate early detection of any SE incursions in NSW, and for ongoing assessment of the risk of SE becoming endemic in the Australian poultry industry. Since SE infection (including PT12 isolated from the 2018–2020 outbreak) does not usually cause clinical signs in infected birds [31], widespread nondiscriminatory surveillance testing of poultry farms is recommended. In addition, a National Foodborne Illness Reduction Strategy (2018–2021+) has been developed to combat human salmonellosis in Australia caused by both ST and non-ST serovars. This strategy highlights the need for action in five core areas including monitoring and surveillance across the most relevant parts of the food supply chain, in particular the poultry and egg industry. The strategy also identifies the need for additional research and development to reduce foodborne illnesses in Australia [51]. Vaccination of poultry against SE is an area currently being explored; no SE vaccine is currently available in Australia for use in any species, although the efficacy of autogenous SE vaccines made with the causal organism isolated from infected farms is currently being investigated by researchers as a potential public health measure [52].

For testing by both qPCR and bacterial culture, both environmental samples and animal samples produced SE-positive results. Interestingly, four SE-infected properties in the described outbreak only tested qPCR-positive with environmental sample testing and would have been incorrectly classified as SE-negative if animal samples alone were tested. *Salmonella* is known to persist and survive in the environment, with one environmental SE isolate remaining viable in an aquatic environment for as long as 60 days after depletion of nutrients [53]. Given this, perhaps other environmental possible sources of SE transmission between properties should also be routinely monitored, for example qPCR testing of dirt and dust collected from the wheels and surfaces of transportation vehicles. Regardless, the success of environmental sampling in this study supports the noninvasive and welfare-friendly environmental sampling strategy outlined in the SE Control Order for ongoing SE surveillance of properties in NSW, Australia, and highlights the importance of ensuring a thorough decontamination of properties following SE incursions [32].

Feces was the most useful animal sample type tested, responsible for 20/37 and 9/22 SE-positive animal samples by qPCR and culture testing, respectively. This result is perhaps not surprising, given the fecal-oral transmission of *Salmonella* spp. However, it serves as a reminder that biosecurity measures against SE need to focus on preventing fecal contamination between sheds and properties, since feces is easily carried on fomites. The high number of SE-positive boot swab samples (*n* = 21 by qPCR testing, *n* = 25 by bacterial culture) in the current study, likely due to poultry fecal contamination, highlights this likely route of transmission and supports the instruction in the SE Control Order to provide boot scrapers, footbaths, or clean “shed boots” [32]. Fecal material can also be transferred onto eggshells, leading to horizontal transmission between birds, as well as possible consumer exposure. Some *Salmonella* serovars (including ST and SE) can survive on eggshell for several weeks and form biofilms [54, 55]. During the outbreak reported in this study, 8/37 and 6/22 egg samples were SE-positive by qPCR and culture testing, respectively. Interestingly, non-SE *Salmonella* serovars were also detected by qPCR in three egg samples; however, only one was able to be cultured but was not sent for serotyping. SE is also transmitted transovarially, thus a proportion of eggs are contaminated internally [14, 56]. Ingestion of contaminated eggs and chicken meat is the major vehicle for human infection with SE, with undercooked eggs and egg-based products most associated with foodborne SE-induced salmonellosis [10–12, 57–59]. In summary, given these results, it seems prudent for both SE surveillance and future disease outbreak investigations to continue testing a mix of both environmental and animal (poultry) samples, including boot covers, surface swabs, feces, and eggs.

Rodents are known as a reservoir of SE and likely contribute to outbreaks by shedding the organism into the poultry farm environment via feces. Various wildlife and free ranging animals have been known to harbor different serovars of *Salmonella* and transmit infection to production animals and humans [60–62]. In the United Kingdom (UK), wildlife from known SE-infected poultry farms were tested, with SE isolated from mouse and rat feces, mouse carcasses, fox feces, and wild bird feces (PT6) [63]. Another study used molecular fingerprinting evidence to suggest the contribution of several wildlife vectors (mice, rats, flies, litter beetles and foxes) to SE maintenance on farms [64]. High wildlife activity around production animals, and particularly intensive layer systems, should therefore be monitored and considered a potential source of infection outbreaks in NSW and elsewhere. In the current study, both SE and non-SE *Salmonella* were detected in rodent carcasses, suggesting that ongoing active testing on non-standard sample types such as rodent feces could be beneficial in monitoring for routes of transmission between properties.

Results from the investigation reported here demonstrate that molecular testing is suitable for SE screening and detection in outbreak situations (qPCR sensitivity 100% and specificity 94.1%), with bacterial culture reserved for confirmatory and viability testing only [65]. Importantly, none of the 10 SE qPCR-negative farms that also had culture performed on all samples were SE culture-positive (i.e., no false-negative results were obtained with SE qPCR testing). Ideally, we would have performed bacterial culture on every sample to allow a complete comparison of culture and qPCR results, but the huge volume of samples received with each submission, media availability, budget limitations, and urgency to produce results to help make informed response decisions, made this impossible. We believe the 15 SE qPCR-positive results we obtained without corroborating culture-positive results during the side-by-side comparison testing, using a qPCR assay developed and well-validated for detection of *Salmonella* spp. in food preparation environments [28] and verified as highly specific (100%) in our laboratory, most likely represented the PCR detection of non-viable organisms or difficulty in isolating SE from culture when other *Salmonella* serovars were present. Morphologically, SE and non-SE *Salmonella* isolates appear identical on a culture plate, and testing of a random selection of suspect *Salmonella* colonies (usually up to five per sample) was undertaken. For the single property that was SE qPCR-positive but SE culture-negative, 30/59 samples were qPCR-positive for *Salmonella* spp., including two environmental swabs that were SE qPCR-positive, suggesting the presence of high levels of competing non-SE *Salmonella*. Despite multiple culture attempts on samples from this property, only 10 different non-SE *Salmonella* serovars were able to be isolated (Alachua, Cerro, Havana, Infantis, Liverpool, Mbandaka, Senftenberg, subsp 1 ser rough:r:1,5, Tennessee, and Typhimurium).

A limitation of qPCR testing was only being able to determine if samples were SE-positive or non-SE *Salmonella* positive, not both, whereas culture has the potential to identify when multiple *Salmonella* serovars, including SE, were concurrently present. However, culture is more laborious, time-consuming, and expensive than high-throughput qPCR testing. The timeframe for reporting qPCR results from receipt of samples in the laboratory in this study was 24–36 h, while the culture-based approach (including presumptive serotyping) produced results approximately 96 h from receipt of samples. Definitive *Salmonella* serotyping results (performed by an external laboratory) usually took approximately another three to 7 days. This significant time difference in receiving results is crucial in the early stages of disease outbreaks and could be the difference between disease elimination and the establishment of SE as an endemic infection in the Australian poultry industry. This time difference highlights that molecular testing is currently the preferred diagnostic tool for rapid screening of many samples over bacterial culture, particularly in an outbreak situation.

Repeat qPCR testing due to inhibition or inconclusive results was uncommonly required (8% of all samples tested), and when it occurred it most frequently involved egg and fecal samples (33% of all egg samples tested, 14% of all fecal samples tested). This was unsurprising, given there are several reports of egg yolk components, fats and proteins, and feces from multiple animal species, causing PCR inhibition [66–69]. When PCR inhibition occurred with incubated BPW testing, qPCR testing was performed on the incubated RVS culture samples, with no repeat extractions of the RVS required (i.e., qPCR testing of RVS samples was 100% successful). Given these results, we recommend qPCR testing of BPW samples to be used as a screening tool to enable rapid reporting of most results in the event of an outbreak situation (in this investigation 92%). In addition, it is recommended to preemptively continue with RVS enrichment from BPW for all egg and fecal samples immediately after the initial 18 h incubation, in case confirmatory testing of SE qPCR-positive samples by bacterial culture is required. As was the case in this study, RVS is typically the preferred broth for the selective enrichment of *Salmonella* from poultry samples [70, 71]. RVS broth is also useful for recovery of atypical *Salmonella* strains from poultry products (e.g., serovars Gallinarum and Pullorum, both of which are currently exotic to Australia) [72].

In total, 34 different *Salmonella* serovars (including SE) were isolated during this outbreak. Since not all *Salmonella* isolates were pursued and serotyped, the number of different serovars present on farms was likely even higher. Over 60 different *Salmonella* serovars have been isolated from a farm environment in NSW, Australia [73]. Other (non-SE) serovars of *Salmonella* isolated during the outbreak investigation included serovars commonly linked to cases of human salmonellosis in NSW including Infantis (6 different properties), Typhimurium (2 properties), Anatum (1 property), Birkenhead (1 property), Virchow (1 property), and Wangata (1 property) [23]. In a study of eggs collected from 26 commercial flocks in Sydney, NSW, Infantis was the most common *Salmonella* serovar isolated from unwashed and uncracked shells [74]. A review of 166 egg-associated foodborne outbreaks of human salmonellosis in Australia between 2001 and 2011 reported that 90% were caused by ST [75]. To our knowledge, this is the first report of *Salmonella* Wangata being isolated from a poultry farm in Australia. *Salmonella* Wangata was cultured from four samples from the same property towards the end of the 2018–2020 outbreak, with all four environmental samples collected from the same shed. Previously in Australia, the source of *Salmonella* Wangata in human infections could not be identified, with environmental contamination due to reservoir maintenance in wildlife populations suspected [76, 77]. Results from this study could help fill this knowledge gap, and therefore ongoing surveillance of non-SE *Salmonella* serovars present on poultry farms in Australia is warranted.

WGS methods provide unprecedented discrimination of *Salmonella* strains and have become established as important tools in both public health and animal disease outbreak investigations internationally. Here, we established that the sequence type 11, Clade B SE isolates sourced from the 18-month outbreak in NSW, Australia in 2018–2020 were all linked by WGS to a single infection source using established criteria for defining *Salmonella* outbreak clusters (i.e., all isolates represented a monophyletic clade) [46, 78]. The observed maximum of four core-genome SNPs across the 18-month interval is consistent with previously observed slow mutation rates observed in *Salmonella* [79, 80]. Interestingly, PT did not confirm this result, with at least three phage types (PTs) found to be present within the WGS-determined outbreak cluster. Bacteriophage typing subdivides *Salmonella* enterica strains (and other bacteria) into epidemiologically significant subgroups based on lysis to a homologous typing phage and resistance to a heterologous typing phage [81, 82]. Different *Salmonella* PTs, however, can have the same clonal genotype, which appears the case in this outbreak [83, 84]. This finding is consistent with previous studies of SE where limited relationships between PT and WGS results were observed [24, 85]. While WGS is widely considered to be more discriminatory than PT, it is important to note that multiple PTs identified within a single WGS outbreak cluster may not necessarily indicate separate subclusters or be a reliable indicator of whether outbreak-sourced isolates are of the same cluster.

## 5. Conclusion

It is unclear why, until recently, Australia's poultry industry has been relatively clear of SE compared to Asia, Europe, the UK, and the USA. Given the absence of clinical signs in poultry with SE infections, it would be of great concern to public health in Australia if SE were to become endemic in layer flocks in the future. This recent SE outbreak in layer farms in NSW (2018–2020) is proof that this concern is valid and is unlikely to be an isolated event. The methods outlined in this report for rapid detection of SE in both environmental and animal samples, with a focus on rapid, high-throughput molecular testing, provide a roadmap for ongoing surveillance activities and testing in future disease outbreaks. Continued serovar identification and tracing of human cases of salmonellosis, and ongoing surveillance and routine testing of animal and environmental samples in the poultry industry, will be required if locally acquired SE infection is to be avoided as a future public health concern in NSW.

## Figures and Tables

**Table 1:** Sample types received at the Elizabeth Macarthur Agricultural Institute (EMAI) for testing.

Environmental	Animal (poultry)
Boot covers*⁣*^*∗*^	Eggs*⁣*^*∗*^
Surface swabs*⁣*^*∗*^	Feces*⁣*^*∗*^
Dirt/soil	Organ swabs
Dust	Cloacal swabs
Water	Blood swab
Poultry feed	—
Egg cartons	—
Foot bath sanitizer	—
Rodent carcass (mummified)	—
Rodent/cockroach feces	—

*⁣* ^*∗*^Routine samples received for culture and molecular testing.

**Table 2:** Total number and proportion of *S. enterica* serovars identified from serotyping during the outbreak in New South Wales (NSW).

Serovars (serotypes)	Total no.	No. properties isolated from	% of total isolates
Alachua	10	1	2.6
Anatum	1	1	0.3
Bahrenfield	1	1	0.3
Bareilly	25	3	6.6
Birkenhead	13	1	3.4
Cerro	5	2	1.3
Chailey	1	1	0.3
Derby	5	2	1.3
Enteritidis (subsp. 1 ser 9,12:g,m:-)	104	12	27.3
Havana	1	1	0.3
Hessarek	1	1	0.3
Hvittingfoss	1	1	0.3
Infantis	74	6	19.4
Liverpool	30	2	7.9
Livingstone	3	1	0.8
Mbandaka	28	5	7.3
Meleagridis	1	1	0.3
Muenster	2	1	0.5
Orion	2	1	0.5
Salmonella subsp. 1 ser 6,7:r:-	1	1	0.3
Salmonella subsp. 1 ser 16:1,v:	2	1	0.5
Salmonella subsp. 1 ser 3,19:-:- (nonmotile strain)	1	1	0.3
Senftenberg	3	1	0.8
Singapore	4	2	1.0
Stanley	1	1	0.3
Subsp. 1 (not SE)	3	2	0.8
Subsp. 1 ser 4,12:-:1,2	1	1	0.3
Subsp. 1 ser rough:r:- (not SE)	1	1	0.3
Subsp. 1 ser rough:r:1,5	11	1	2.9
Subsp. 3*⁣*^*∗*^	1	1	0.3
Tennessee	1	1	0.3
Typhimurium	38	2	10.0
Virchow	1	1	0.3
Wangata	4	1	1.0

Total	381	N/A	100%

*Note:* From a total of 23 properties that had bacterial culture performed, 409 *Salmonella* isolates were cultured from 22 different properties, with 381 isolates were sent for serotyping. Serotyping was performed by the Australian Salmonella Reference Centre, Institute of Medical and Veterinary Science, Adelaide, and the NSW Enteric Reference Laboratory, Westmead.

Abbreviation: N/A, not applicable.

*⁣* ^*∗*^Isolated from a rodent carcass.

**Table 3:** Results from SE phage identification (*n* = 16).

Date of submission	Property	Sample type	Phage result
January 2019	Property 2	Eggs (A) Eggs (A)	RDNC*⁣*^*∗*^ 12

April 2019	Property 10	Feed composite station (E)	12

May 2019	Property 16	Shed slats (E)	12

February 2020	Property 25	Rodent bait station (E) Eggshells from shed (A)	1b 1b

March 2020	Property 6	Boot swab from poultry shed (E) Feed tray (E) Feces - poultry (A) Egg cool room trolley in shop (E) Water trough/drinking nozzle (E) Feces - poultry (A) Boot swab outside next to pile of cow manure (E) Chemical fridge (E)	1b 1b 1b 1b 1b 1b 1 b 1b

March 2020	Property 24	Grading room cool room (E) Candling station (E)	12 1 b

*Note:* SE isolates from six different properties, submitted during different months of the 2018–2020 outbreak, were tested. Each month represents isolates collected from one individual property. Phage testing was performed by the Australian Salmonella Reference Centre, Institute of Medical and Veterinary Science, Adelaide.

Abbreviations: A, animal (poultry) sample; E, environmental sample.

*⁣* ^*∗*^Phage type RDNC refers to isolates with lytic patterns that do not conform to a recognized phage type.

**Table 4:** Comparison of qPCR and bacterial culture results for SE testing from nine different New South Wales (NSW) poultry properties (*n* = 296).

	SE culture −ve	SE culture +ve	Total
SE qPCR −ve	238	0	238
SE qPCR +ve	15	43	58

Total	253	43	296

*Note:* SE-positive samples by qPCR testing had amplification in all three targets (*invA*, *prt* and *Sdf-1*). Suspect SE-positive isolates from bacterial culture (based on morphology on *Salmonella* selective media, MALDI-ToF MS and supplementary biochemical testing) were confirmed by the Australian Salmonella Reference Centre, Institute of Medical and Veterinary Science, Adelaide or the NSW Enteric Reference Laboratory, Westmead. Results from samples not deliberately tested side-by-side (i.e., culture performed to confirm a qPCR-positive result or to investigate a qPCR-inconclusive result) were excluded from analyses to remove testing bias. For this reason, there are less SE qPCR-positive/culture-positive results available for comparison testing (*n* = 43) than SE isolates obtained during the outbreak (*n* = 104; Table 2), despite qPCR testing being performed on every sample submitted.

Abbreviations: +ve, positive; −ve, negative; MALDI-ToF MS, matrix-assisted laser desorption/ionization-time of flight mass spectrometry.

## Data Availability

The data that support the findings of this study are available from the corresponding author upon reasonable request.

## Supporting Information

Supporting Information 1 Table S1. In-house verification of the published qPCR SE assay used in this study was performed using DNA extracted from a total of 76 isolates, including 46 SE isolate isolates (of which 4 were human isolates from this outbreak and 2 were Clade A isolates), 23 non-SE *Salmonella* serovars, and seven isolates representing other Enterobacterales. D, detected; ND, not detected; qPCR, quantitative real-time PCR; SE, *S. enterica* serovar Enteritidis.

Supporting Information 2 Table S2. Number of positive samples for non-SE *Salmonella* and SE during the 2018−2020 outbreak by sample type, as tested by qPCR and bacterial culture. qPCR was performed on all samples; however, bacterial culture was only performed on select samples. SE, *S. enterica* serovar Enteritidis; qPCR, quantitative real-time PCR.

Supporting Information 3 Table S3. Serovar (serotype) results when different *Salmonella* serovars were isolated from the same sample. For submissions from six different properties, multiple (i.e., two or more) *Salmonella* colonies isolated from the same sample were serotyped on 29 occasions, with different serovars identified in 13/29 (45%) of samples from two properties. Most of the samples for which multiple *Salmonella* colonies were sent for serotyping were submitted from the same property (22/29; Property 3). E, environmental.

Supporting Information 4 Table S4. Testing details for the 15 discordant results (SE qPCR-positive, SE culture-negative). −, negative; +, positive; A, animal (poultry); E, environmental; qPCR, quantitative real-time PCR; SE, *S. enterica* serovar Enteritidis.

Supporting Information 5 Table S5. WGS analysis was performed during the New South Wales (NSW) 2018−2020 outbreak by EMAI on 32 SE isolates. In total, 15/82 (18%) environmental-derived SE isolates and 5/22 (23%) animal-derived isolates in this study, obtained from 9 (of 12) different SE culture-positive properties, had WGS performed. Three SE culture-positive properties (Property 5, Property 17, Property 22) did not have WGS performed on any of the 11 SE isolates obtained from these properties. Additionally, 12 SE isolates obtained from other laboratories during the outbreak had WGS performed. The index SE isolate sourced from frozen meringue cake (18-9539-0146) is not shown. EMAI, Elizabeth Macarthur Agricultural Institute (NSW Department of Primary Industries and Regional Development); SE, *S. enterica* serovar Enteritidis; WGS, whole genome sequencing.
