# Supplementary material for: Application of a Quantitative Real-Time PCR Assay for Early Detection of Salmonella enterica Serovar Enteritidis on Poultry Farms During an Outbreak in New South Wales, Australia (2018–2020)
Source: Transbound Emerg Dis. 2025 Jun 4;2025:9937941. doi: 10.1155/tbed/9937941 (PMC12158595; doi:10.1155/tbed/9937941)
Supplement: Supporting Information 1 — Table S1. In-house verification of the published qPCR SE assay used in this study was performed using DNA extracted from a total of 76 isolates, including 46 SE isolate isolates (of which 4 were human isolates from this outbreak and 2 were Clade A isolates), 23 non-SE Salmonella serovars, and seven isolates representing other Enterobacterales. D, detected; ND, not detected; qPCR, quantitative real-time PCR; SE, S. enterica serovar Enteritidis. [file 9937941.f1.docx]

| **Isolate (*n* = 76)** | **qPCR result** |
| --- | --- |
| 46 x SE | 44 D, 2 ND (both Clade A isolates) |
| 23 x non-SE serotypes  Anatum  Birkenhead  Boreilly  Bovismorbificans  Bredeney  Cerro  Chester  Dublin x 2  Florida  Give  Infantis  Mbandaka  Newport  Reading  Rough strain *Salmonella*  Saintpaul  Subsp. ser 4:12:I-  Tennessee  Typhimurium  Warragul  3B ser 47:K:2  3A ser 44:24,223 | 23 ND |
| 7 x Enterobacterales  *Citrobacter braakii*  *Citrobacter freundii*  *Escherichia coli*  *Shigella* spp.  *Yersinia enterocolitica*  *Yersinia pseudotuberculosis* x 2 | 7 ND |
