# Supplementary material for: Application of a Quantitative Real-Time PCR Assay for Early Detection of Salmonella enterica Serovar Enteritidis on Poultry Farms During an Outbreak in New South Wales, Australia (2018–2020)
Source: Transbound Emerg Dis. 2025 Jun 4;2025:9937941. doi: 10.1155/tbed/9937941 (PMC12158595; doi:10.1155/tbed/9937941)
Supplement: Supporting Information 2 — Table S2. Number of positive samples for non-SE Salmonella and SE during the 2018−2020 outbreak by sample type, as tested by qPCR and bacterial culture. qPCR was performed on all samples; however, bacterial culture was only performed on select samples. SE, S. enterica serovar Enteritidis; qPCR, quantitative real-time PCR. [file 9937941.f2.docx]

| **Samples received** | **qPCR testing** | | | **Bacterial culture** | | |
| --- | --- | --- | --- | --- | --- | --- |
|  | **Non-SE *Salmonella*** | **SE** | **Total tested** | **Non-SE *Salmonella*** | **SE** | **Total tested** |
| **Environmental samples (*n* = 1609)**  Surface swabs  Boot covers  Dirt/soil  Dust  Water  Chicken Feed  Egg cartons  Foot bath sanitiser  Rodent carcass  Rodent/cockroach faeces | 140  99  1  10  0  2  0  0  2  0 | 126  21  7  0  2  0  0  0  0  1 | 1300  219  28  12  12  26  1  7  3  1 | 167  59  9  4  0  2  0  0  1  0 | 55  25  1  0  0  0  0  0  0  1 | 422  109  15  4  1  4  1  1  3  2 |
| SUBTOTAL | 254 | 157 | 1609 | 242 | 82 | 562 |
| **Poultry (animal) samples (*n* = 449)**  Pooled eggs  Faeces  Cloacal swabs  Organ/blood swabs | 3  17  0  6 | 8  20  2  7 | 181  145  100  23 | 1  21  0  6 | 6  9  1  6 | 67  62  3  23 |
| SUBTOTAL | 26 | 37 | 449 | 28 | 22 | 155 |
| **TOTAL (*n* = 2058)** | 280 | 194 | 2058 | 270 | 104 | 717 |
