# Supplementary material for: Application of a Quantitative Real-Time PCR Assay for Early Detection of Salmonella enterica Serovar Enteritidis on Poultry Farms During an Outbreak in New South Wales, Australia (2018–2020)
Source: Transbound Emerg Dis. 2025 Jun 4;2025:9937941. doi: 10.1155/tbed/9937941 (PMC12158595; doi:10.1155/tbed/9937941)
Supplement: Supporting Information 3 — Table S3. Serovar (serotype) results when different Salmonella serovars were isolated from the same sample. For submissions from six different properties, multiple (i.e., two or more) Salmonella colonies isolated from the same sample were serotyped on 29 occasions, with different serovars identified in 13/29 (45%) of samples from two properties. Most of the samples for which multiple Salmonella colonies were sent for serotyping were submitted from the same property (22/29; Property 3). E, environmental. [file 9937941.f3.docx]

| **Submission No.** | **Property** | **Sample Type** | **Serovar 1** | **Serovar 2** |
| --- | --- | --- | --- | --- |
| M19-07156/8 | 3 | Tray (E) | Typhimurium | Infantis |
| M19-07156/11 | 3 | Chair (E) | Typhimurium | Infantis |
| M19-07156/28 | 3 | Boot swab - shed (E) | Typhimurium | Liverpool |
| M19-07156/34 | 3 | Boot swab – shed (E) | Alachua | Liverpool |
| M19-07156/36 | 3 | Boot swab – shed (E) | Typhimurium | Subsp. 1 (not SE) |
| M19-07156/40 | 3 | Boot swab – shed (E) | Typhimurium | Havana |
| M19-07156/41 | 3 | Boot swab – shed (E) | Typhimurium | Tennessee |
| M19-07156/45 | 3 | Boot swab – shed (E) | Alachua | Mbandaka |
| M19-07156/54 | 3 | Dust (E) | Typhimurium | Subsp. 1 ser rough:r:1,5 |
| M19-07156/58 | 3 | Trolley (E) | Typhimurium | Infantis |
| M19-07156/59 | 3 | Fan (E) | Typhimurium | Infantis |
| M19-07498/95 | 22 | Rodent carcass (E) | Subsp. 1 ser rough:r:- | Subsp. 3 |
| M20-03792/92 | 6 | Boot swab – cow manure (E) | Enteritidis | Virchow |
