# Supplementary material for: Application of a Quantitative Real-Time PCR Assay for Early Detection of Salmonella enterica Serovar Enteritidis on Poultry Farms During an Outbreak in New South Wales, Australia (2018–2020)
Source: Transbound Emerg Dis. 2025 Jun 4;2025:9937941. doi: 10.1155/tbed/9937941 (PMC12158595; doi:10.1155/tbed/9937941)
Supplement: Supporting Information 4 — Table S4. Testing details for the 15 discordant results (SE qPCR-positive, SE culture-negative). −, negative; +, positive; A, animal (poultry); E, environmental; qPCR, quantitative real-time PCR; SE, S. enterica serovar Enteritidis. [file 9937941.f4.docx]

| **Submission No.** | **Property** | **Sample Type** | **RESULTS** | | |
| --- | --- | --- | --- | --- | --- |
|  |  |  | **SE qPCR** | **SE culture** | **Non-SE culture** |
| M19-04153/196 | 8 | Environmental swab (E) | + | - | - |
| M19-04153/197 | 8 | Environmental swab (E) | + | - | - |
| M19-04153/201 | 8 | Environmental swab (E) | + | - | - |
| M19-04797/35 | 2 | Boot cover (E) | + | - | + (Derby) |
| M19-06922/3 | 16 | Drag swab (E) | + | - | - |
| M19-06922/5 | 16 | Drag swab (E) | + | - | - |
| M19-06922/6 | 16 | Drag swab (E) | + | - | - |
| M19-08475/35 | 5 | Drag swab (E) | + | - | - |
| M20-02756/3 | 25 | Environmental swab (E) | + | - | - |
| M20-02756/8 | 25 | Environmental swab (E) | + | - | - |
| M20-02756/11 | 25 | Environmental swab (E) | + | - | - |
| M20-02756/36 | 25 | Environmental swab (E) | + | - | - |
| M20-02756/40 | 25 | Environmental swab (E) | + | - | - |
| M20-02756/60 | 25 | Faeces (A) | + | - | - |
| M20-02756/64 | 25 | Faeces (A) | + | - | - |
