# Supplementary material for: Application of a Quantitative Real-Time PCR Assay for Early Detection of Salmonella enterica Serovar Enteritidis on Poultry Farms During an Outbreak in New South Wales, Australia (2018–2020)
Source: Transbound Emerg Dis. 2025 Jun 4;2025:9937941. doi: 10.1155/tbed/9937941 (PMC12158595; doi:10.1155/tbed/9937941)
Supplement: Supporting Information 5 — Table S5. WGS analysis was performed during the New South Wales (NSW) 2018−2020 outbreak by EMAI on 32 SE isolates. In total, 15/82 (18%) environmental-derived SE isolates and 5/22 (23%) animal-derived isolates in this study, obtained from 9 (of 12) different SE culture-positive properties, had WGS performed. Three SE culture-positive properties (Property 5, Property 17, Property 22) did not have WGS performed on any of the 11 SE isolates obtained from these properties. Additionally, 12 SE isolates obtained from other laboratories during the outbreak had WGS performed. The index SE isolate sourced from frozen meringue cake (18-9539-0146) is not shown. EMAI, Elizabeth Macarthur Agricultural Institute (NSW Department of Primary Industries and Regional Development); SE, S. enterica serovar Enteritidis; WGS, whole genome sequencing. [file 9937941.f5.docx]

| **Submission No.** | **Property** | **Date of submission** | **Sample type** |
| --- | --- | --- | --- |
| SE isolates cultured at EMAI (*n* = 20): | | | |
| M19-00461-16A1* | 2 | January 2019 | Eggs |
| M19-00461-17A1* | 2 | January 2019 | Eggs |
| M19-04153-204H3 | 8 | March 2019 | Cockroach/mouse faeces |
| M19-04797-16B | 2 | March 2019 | Environmental swab (candling area) |
| M19-04950-16M2* | 10 | April 2019 | Environmental swab (feed composite station) |
| M19-05410-47A | 1 | April 2019 | Environmental swab (conveyer in main shed) |
| M19-06375-20A | 14 | May 2019 | Environmental swab (grading window ledge) |
| M19-06922-1A2* | 16 | May 2019 | Environmental swab (shed slats) |
| M20-02756-42A* | 25 | February 2020 | Environmental swab (rodent bait station) |
| M20-02756-48A* | 25 | February 2020 | Eggshells |
| M20-02902-17A* | 6 | March 2020 | Environmental swab (boot swab from shed) |
| M20-02902-51A* | 6 | March 2020 | Environmental swab (feed tray) |
| M20-02902-80A* | 6 | March 2020 | Faeces (poultry) |
| M20-03161-8B* | 6 | March 2020 | Environmental swab (egg cool room trolley in shop) |
| M20-03792-57D* | 6 | March 2020 | Environmental swab (water trough/drinking nozzle) |
| M20-03792-72A* | 6 | March 2020 | Faeces (poultry) |
| M20-03792-92F* | 6 | March 2020 | Boot swab (next to pile of cow manure pile) |
| M20-03792-112B* | 6 | March 2020 | Environmental swab (chemical fridge) |
| M20-02976-3A* | 24 | March 2020 | Environmental swab (grading room cool room) |
| M20-02976-22A* | 24 | March 2020 | Environmental swab (candling station) |
| SE isolates sent to EMAI for WGS (*n* = 12):^#^ | | | |
| M19-00480 | N/A | January 2019 | 3 isolates submitted by another laboratory |
| M19-00959 | N/A | January 2019 | 8 isolates submitted by another laboratory |
| M19-04700 | N/A | March 2019 | 1 isolate submitted by another laboratory |

* These 16 SE isolates also had phage identification testing performed (Table 3).

^#^ These 12 SE isolates were provided during the outbreak by other laboratories for confirmatory testing and sequencing since SE is a notifiable disease in NSW. They were excluded from other analyses in this manuscript because no initial qPCR or culture testing was performed at EMAI.
